# P-1066. Microbiological profile of nosocomial pneumonias in the icu of a third level public health center of the dominican republic

**DOI:** 10.1093/ofid/ofaf695.1261

**Published:** 2026-01-11

**Authors:** David De Luna, Yori A Roque, Alfredo J Mena Lora, Jose Esteban Tejada

**Affiliations:** Hospital Metropolitano de Santiago, Santiago, Santiago, Dominican Republic; Hospital Metropolitano de Santiago (HOMS), Santiago, Santiago, Dominican Republic; University of Illinois Chicago, Chicago, Illinois; Hospital Jose Maria Cabral y Baez, Santiago, Santiago, Dominican Republic

## Abstract

**Background:**

- Hospital-acquired pneumonia (HAP) accounts for approximately 22% of all hospital-acquired infections, with an attributable mortality rate reaching up to 33% in critically ill patients.
- HAP increases length of stay, broad-spectrum antibiotic use, costs, and complications, negatively affecting patient outcomes

**Figure 1.**
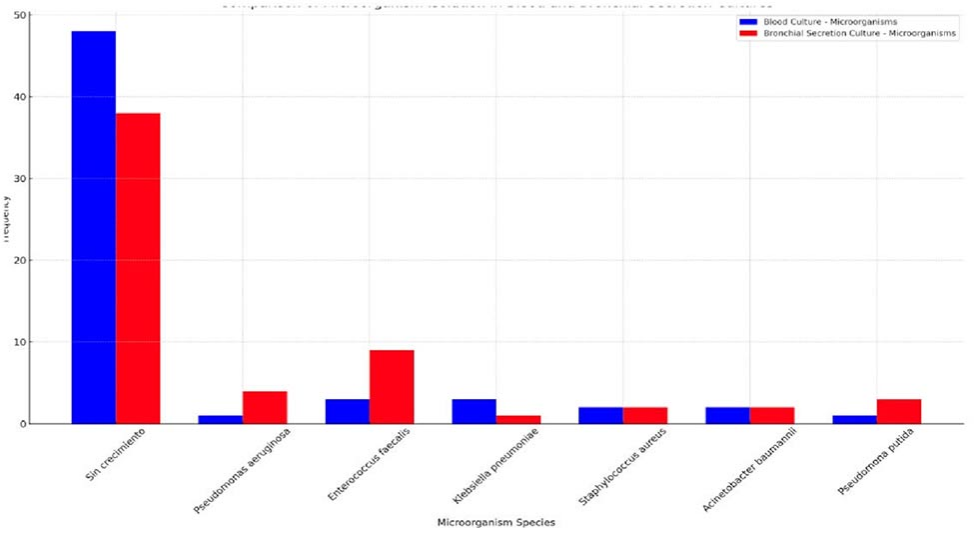
Species of microorganism isolated in blood culture and culture of bronchial secretion

**Methods:**

A retrospective cross-sectional observational study was conducted to analyze the microbiological profile and clinical outcomes of patients diagnosed with HAP/VAP during the period from July 1, 2023, to July 1, 2024.

- HAP and VAP were defined using IDSA guideline criteria, with HAP in those hospitalized >48 hours, and VAP >48 hours of mechanical ventilation
- Patients were required to have at least one culture report from blood or bronchial aspirate obtained via tracheal cannula (MiniBAL).
- Statistical analysis was performed using the Chi-square test (χ²)and ANOVA for variable cross-analysis

**Figure 2.**
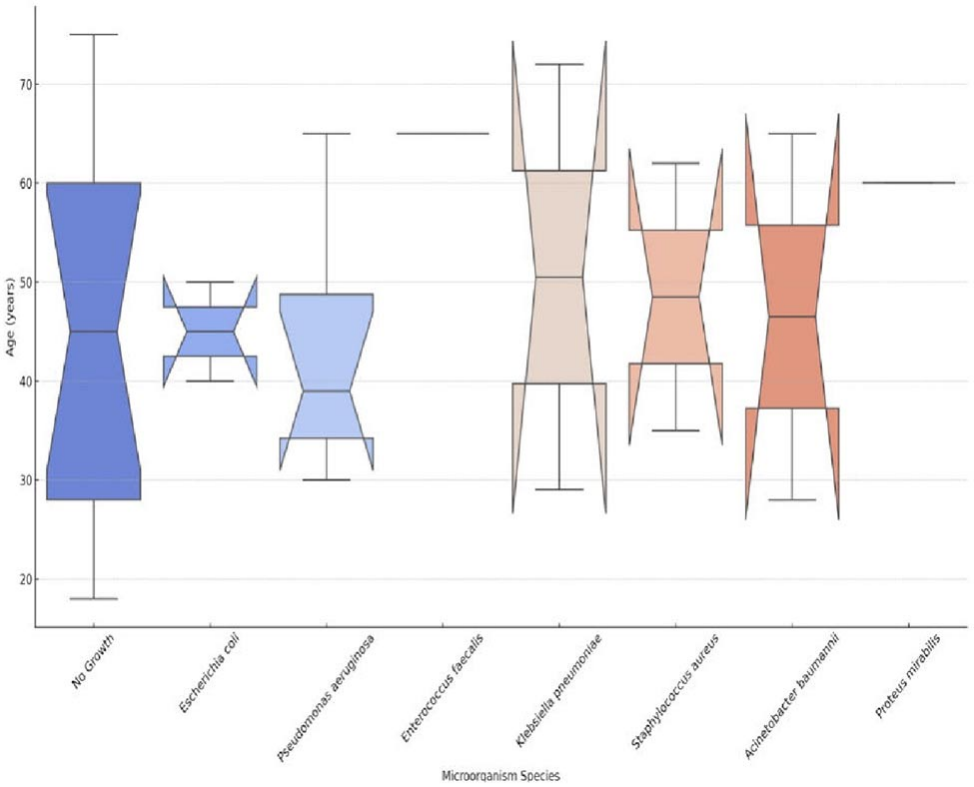
Association between group age and bacterial isolation on bornchial culture

**Results:**

Of a total of 270 records analyzed, only 60 patients met the inclusion criteria, of which 42 (70%) were male;

- The average age was 44.5 +/- 18.42 years, with the range of 18-29 being the most frequent (30%). 60% of the patients had an associated co-morbidity, HTN (30%) followed by DM (23.3%), were the most frequent.
- The average LOS was 22.5 (+/- 9.46) days, where 91.7% of patients were ventilated
- Regarding cultures, 63.3% reported no growth, with P. auriginosa being the most frequent (15%), followed by E. coli (6.7%) and A. baumanii (5%). (Figure 1), we found a statistical relationship between the age groups and the isolated microorganism (p=0.025).
- During septic shock events, microorganisms were isolated more frequently, with P. auriginosa and E. Coli being the most frequent, in 7 and 3 cases, respectively, we did not find a difference between survival and cultures reported without growth (P value 0.334). (Figure 2)

**Conclusion:**

In this study, lack of culture growth was not associated with better survival

- Critical illness with Septic shock had higher likelihood of culture positivity
- These findings reinforce the importance of monitoring specific complications according to age groups and the identified microorganism to improve clinical outcomes.

**Disclosures:**

All Authors: No reported disclosures

